# Coeliac disease patients do not produce antibodies to a common cerebellar epitope

**DOI:** 10.1186/s40673-014-0018-3

**Published:** 2014-12-14

**Authors:** Volga Tarlac, Louise Kelly, Robert P Anderson, Nicole Bye, Elsdon Storey

**Affiliations:** Department of Medicine (Neuroscience), Monash University, (Alfred Hospital Campus), Commercial Road, Melbourne, VIC 3004 Australia; ImmusanT, Inc. One Kendall Square, Suite B2004, Cambridge, MA 02139 USA; The Alfred Hospital, National Trauma Research Institute, Melbourne, VIC 3004 Australia

**Keywords:** Gluten ataxia, Coeliac disease, Ataxia, Purkinje cell, Cerebellum, Anti-gliadin

## Abstract

**Background:**

Most adult-onset sporadic ataxias are unexplained, and the claim that many of these may be a result of gluten sensitivity has led to uncertainty as to whether to test for anti-gliadin antibodies (αGAb) and, if present, whether to recommend a gluten-free diet or continue searching for other causes of ataxia. This uncertainty arises in part from the frequency of αGAb in the population (about 1 in 10), but recent work delineating transglutaminase 6 as the target antigen in gluten ataxia has clarified the situation somewhat. Our aim was to determine whether there is molecular mimicry between cerebellar Purkinje cell antigens and gluten in subjects selected for recent diagnosis of CD rather than for ataxia.

**Results:**

High titre αGAb sera from 11 newly-diagnosed CD patients and normal sera from 10 healthy controls were used to detect cross-reacting antibodies to cerebellar and cerebral cortex antigens in mouse, monkey and human tissue. None of the CD patients displayed ataxia. Mouse and human cerebellar and cerebral cortex extracts were analysed by Western blot probed with CD and control sera. Immunofluorescence microscopy was used on mouse and monkey cerebellar sections immunostained with CD and control sera to detect cross-reacting IgG antibodies.

Western blot analysis of cerebellar and cerebral cortex extracts probed with CD sera did not demonstrate any specific immunoreactivity unique to the cerebellum. An identical twin pair with CD produced different patterns of reactivity. Immunofluorescence staining of mouse and monkey cerebellar sections showed most control and CD sera reacted non-specifically, with the exception of two CD and one control sera, each having a unique staining pattern.

**Conclusions:**

CD patient sera with high titre αGAb do not detect a common Purkinje cell or cerebellar-specific epitope. The pattern of reactivity is not solely dependent on genetic background.

## Background

Classical coeliac disease (CD) is a chronic immune-mediated enteropathy, triggered by the ingestion of gluten proteins from wheat, rye and barley in genetically susceptible individuals positive for HLA DQ2 and/or DQ8, and resulting in malabsorption [1,2]. Non-classical CD is increasingly recognised, and consists of enteropathy without malabsorption [2]. Gliadin is a moiety of wheat prolamins, and IgA anti-gliadin antibodies have been used to support the diagnosis of CD. They are, however, relatively non-specific, being found in about 1 in 10 individuals in Anglo populations [3], compared with a prevalence of classical plus non-classical CD of about 1 in 100 in the USA [4]. Recent Australian figures are similar, showing αGAb IgG in 17% and αGAb IgA in 7%, compared with an estimated prevalence of coeliac disease 1.6% in a random community sample [5].

Gliadin is deamidated in the gut wall by tissue transglutamidase (transglutamidase-2 – TG2) and this deamidated protein stimulates a cellular immune response in HLA DQ2/8 individuals [6]. Antibodies to deamidated gliadin peptide, to TG2, and anti-endomysial antibodies are now used to support the diagnosis of CD, being much more specific than anti-gliadin antibodies [2]. However, diagnosis of CD in adults and most children still requires confirmatory small bowel biopsy [2,7].

An area of recent interest has been the relationship between gluten sensitivity and (otherwise) idiopathic sporadic cerebellar ataxia. Gluten ataxia (GA) is currently defined as (otherwise) idiopathic sporadic ataxia with anti-gliadin antibodies [2]. The presentation is usually as a pure ataxia of insidious onset with or without an accompanying sensory-motor, length-dependent neuropathy, although a subacute course, myoclonus, opsoclonus and palatal tremor are all described [1].

Most but not all studies have shown a high prevalence of gluten sensitivity in sporadic ataxia patients eg. [3,6]. There is only partial overlap with CD, with only about one third having enteropathy, and less than 40% having anti-TG2 antibodies [1]. Conversely, ataxia and/or neuropathy are found in about 6-10% of CD patients [7,8]. This incomplete overlap has recently been illuminated by the finding that antibodies to TG-6 (especially IgG) are found in about 75% of patients with GA [9]. TG-6 is a predominantly neural isoform of transglutaminase, and is found extracellularly as well as intracellularly [10]. The extracellular location implies that it might be a pathogenic target of humoral autoimmunity, and indeed an anti-TG2 antibody cross-reactive with TG-3 and TG-6 induced transient ataxia in mice when injected intraventricularly [11]. Moreover, a mutation in TG-6 has been reported to cause dominantly-inherited SCA 35 in three Chinese families with three different heterozygous mutations [12,13], emphasising the potential for TG-6 as a pathogenic target in GA.

GA does remain somewhat contentious, however. In part, this may arise from the use of the relatively non-specific anti-gliadin antibodies to define the syndrome. Furthermore, some studies have failed to show an increased rate of anti-gliadin antibodies in sporadic ataxia [14]. Moreover, patients with hereditary ataxias have been reported to have higher rates of anti-gliadin positivity than healthy controls [15]. This raises the possibility that anti-gliadin antibodies might be epiphenomena, being generated against cross-reactive cerebellar epitopes unmasked by cerebellar damage from other causes, in genetically-predisposed individuals. It is also possible that cerebellar damage from other causes might expose neo-epitopes on cerebellar TG-6 itself.

This study aimed to determine whether cerebellar epitopes are recognised by sera from newly-diagnosed CD patients with high-titre anti-gliadin antibodies (αGAb), regardless of the presence or absence of ataxia. This cross-reactivity would support the hypothesis that αGAbs cross-react with cerebellar antigens, and indeed also the converse hypothesis that GA may sometimes be diagnosed in patients in whom cerebellar neo-epitopes are exposed by cerebellar damage from non-gluten sensitivity causes, resulting in antibodies cross-reactive with gliadin. We postulated that an epitope present in cerebellum but not cerebrum would be identified by sera from a subset of CD patients, but not from control subjects.

## Results

### Newly diagnosed CD patients have high titre gliadin antibodies

**Figure 1:**
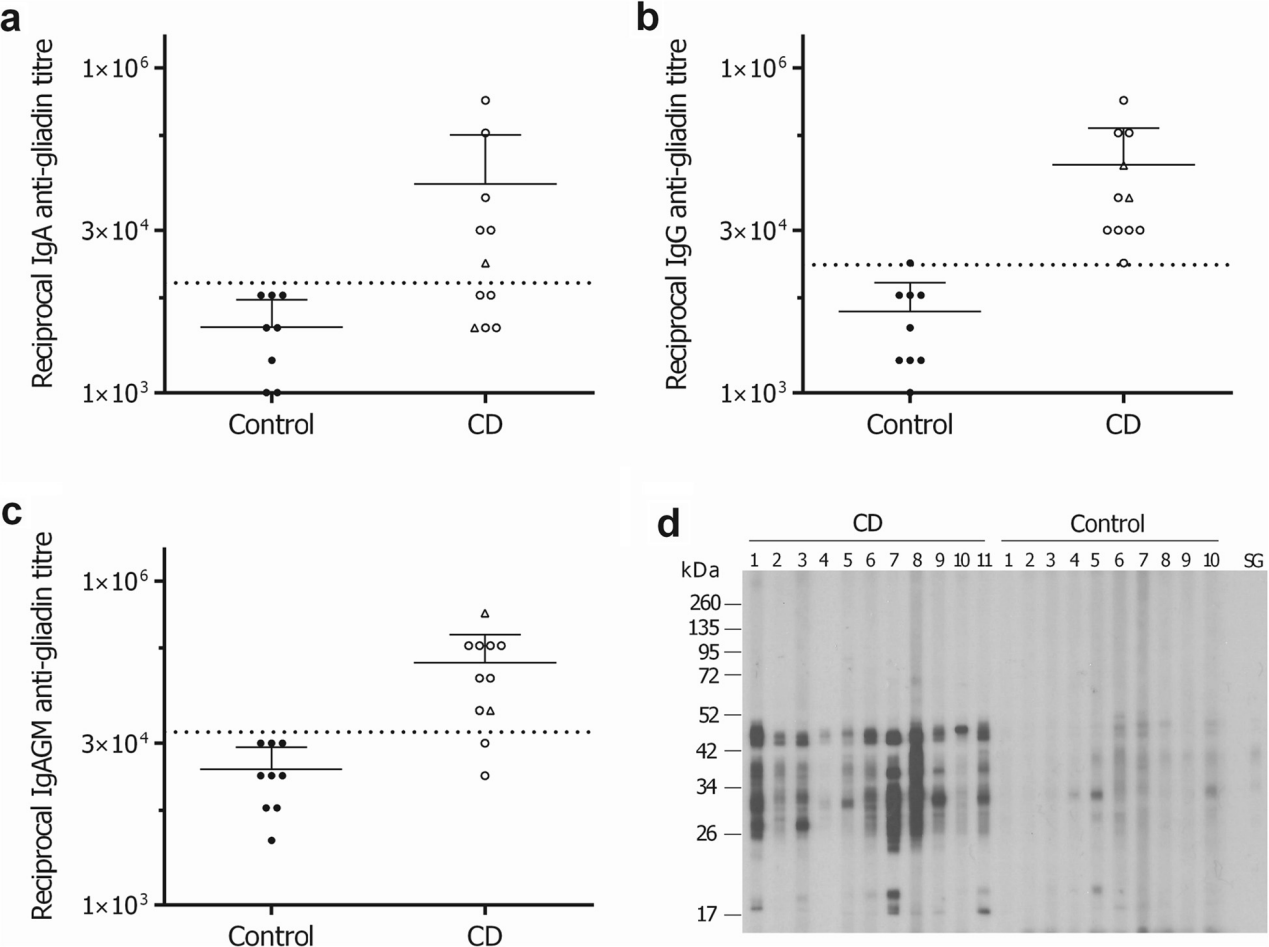
**CD patients have high titres of anti-gliadin antibodies.** The reciprocal titre of anti-gliadin **(a)** IgA, **(b)** IgG and **(c)** IgA,G,M-class antibodies in 11 CD and 9 control subjects was measured by an in-house gliadin ELISA. Error bars represent mean and standard deviation and the horizontal dotted lines indicate 2 standard deviations from the control mean titre. Triangle symbol within graphs denotes twin sibling CD patients. **(d)** Western blot of wheat gliadin extract probed with serum from CD and control patients for IgG antibodies specific to gliadin, and a rabbit anti-gliadin antibody (SG). The CD patients had a strong yet heterogeneous pattern of immunoreactivity to gliadin.

Serum from newly diagnosed CD patients and healthy controls was tested by ELISA for the presence of αGAb. CD patients had high αGAb titres, with mean titres of 1 in 2.15 × 10^4^ (range <1 in 1.0 × 10^3^ to 1 in 1.28 × 10^5^) for IgA (Figure 1a), 1 in 8.4 × 10^4^ (range <1 in 1.6 × 10^4^ to 1 in 2.6 × 10^5^) for IgG (Figure 1b), and 1 in 1.1 × 10^5^ (range <1 in 8.0 × 10^3^ to 1 in 2.6 × 10^5^) for IgA,G and M together (Figure 1c). All 11 (100%) CD patient αGAb IgG titres were >2 SD above the mean of the control titre, compared with only 6/11 (55%) for IgA and 9/11 (82%) for IgA,G and M combined. Patient CD7 had the highest titre of αGAb for all Ig isotypes tested. A pair of identical twins (CD4 and CD5) were also included in this study and their titres are identified in Figure 1a-c with a triangle symbol. Western blot analysis of wheat gliadin extract probed with patient serum (1:250 to 1:1000 dilutions) for αGAb IgG antibodies (Figure 1d) showed a heterogeneous reactivity profile between the CD patients and very little reactivity of serum from the healthy controls. The identical twin patients, CD4 and CD5, had similar IgG immunoreactivity to gliadin.

### Western blot analysis did not reveal a CD-specific cross-reacting cerebellar epitope mouse cerebellum

**Figure 2:**
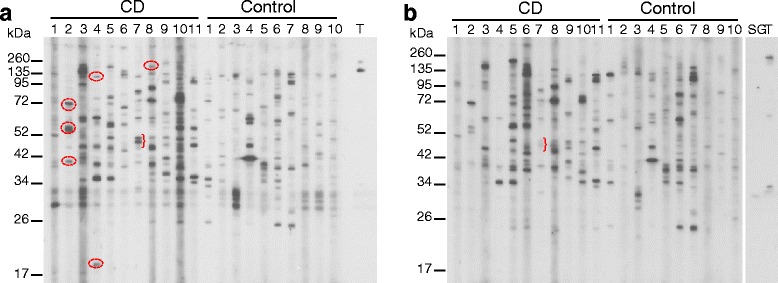
**CD patient serum has a heterogeneous immunoreactivity to mouse brain protein extracts.** Western blots of mouse brain protein extracts probed for cross-reacting antibodies with CD and control patient serum. Mouse cerebellar **(a)** and cerebral cortex **(b)** total protein extracts were probed with CD and control serum for IgG cross-reacting antibodies. Both CD and control serum identified numerous bands of varying size and intensity. The circled bands indicate immunoreactive proteins that are unique to the cerebellum, and identified by CD but not control serum. Bands indicated by parenthesis have markedly less intensity or cerebral cortex Western. CD4 and 5 are the identical twins. TG6 rabbit polyclonal did not detect either the long 79 kDa or short 71 kDa isoform on the two brain regions tested. However, the TG6 antibody detected a band of 170 kDa in the cerebellum and bands of 30–32, 60 and >260 kDa in cerebral cortex extracts. T = anti-TG6 antibody and SG = rabbit anti-gliadin antibody.

Sera from adults with CD and healthy controls were screened for autoantibodies to total protein extracts from adult mouse cerebellum and cerebral cortex by Western blot. Total protein extracts of mouse cerebellum (Figure 2a) and cerebral cortex (Figure 2b) were separated by SDS-PAGE and probed for cross-reacting IgG antibodies. Both CD and healthy control samples produced a complex immunoreactive banding profile. It is notable that CD patients 4 and 5 – identical twins diagnosed within a week of each other – had a similar pattern of gliadin immunoreactivity (Figure 1d), but quite different patterns of cerebellar immunoreactivity. To identify any immunoreactive targets that were unique to mouse cerebellum as distinct from mouse cerebrum, Western blots of mouse cerebellum and cerebral cortex were compared to each other (Figure 2). CD patients 2, 4, and 8 had autoantibodies directed to cerebellar proteins not detected by their sera in the cerebral cortex. CD patient 8 detected a single unique protein of 185 kDa. CD patients 2, 4 and 7 detected multiple targets that were unique to cerebellum or only faintly detected in the cerebral cortex; CD2 detected targets of 40, 55, 58, 70 and 72 kDa; CD4 detected two bands of 20 kDa and 120 kDa; CD7 detected a doublet of 47–50 kDa. The only uniquely cerebellar immunoreactive proteins that were found only in CD patients and not in healthy controls were a 20 kDa protein detected solely in CD4, a 55 kDa protein detected by CD2 and a 185 kDa protein detected by patient CD8. Comparison of the immunoreactive profile of CD and control sera to mouse cerebellum did not identify proteins that were common to CD patients and absent in healthy controls. Table 1 summarises the unique immunoreactive bands. TG6 has two isoforms of 70–71 and 79 kDa [10] but was detected as a faint 60 kDa band in the cerebral cortex only, on Western blot (Figure 2b). The TG6 antibody detected additional bands of 30–32 and >260 kDa in cerebral cortex and bands of 170 kDa and >260 kDa in the cerebellum (Figure 2a and b). None matched the bands detected by the CD patient sera. There was minimal cross-reaction between rabbit polyclonal antigliadin antibody (Sigma) and cerebellar antigens, and none at the Mr’s of TG6.

**Table 1:** **Summary of main findings for Western blotting and immunohistochemistry**

**Subject**	**Mouse tissue Western (kDa)**		**Human tissue Western (kDa)**		**IHC**	
	**Unique to cerebellum**	**Unique to CD patients**	**Unique to cerebellum**	**Unique to CD patients**	**Mouse Cb**	**Monkey Cb**
CD1	-ive	-ive	40	-ive	ns	ns
CD2	70	55	-ive	-ive	granular layer INs	ns
CD3	-ive	-ive	-ive	-ive	ns	ns
CD4*	20 & 120	20	130	–	ns	ns
CD5*	-ive	-ive	-ive	-ive	ns	ns
CD6	-ive	-ive	-ive	-ive	ns	ns
CD7	-ive	-ive	-ive	-ive	granular layer INs	ns
CD8	185	185	34	-ive	ns	ns
CD9	-ive	-ive	-ive	-ive	ns	ns
CD10	-ive	-ive	34 & 130	-ive	nucleoli	nucleoli
CD11	-ive	-ive	-ive	-ive	granular layer Ins	basket cell-like and parallel fibres

ns = non-significant, −ive = no unique band on Western blot, Ins = interneuron, * = identical twin siblings.

**Figure 3:**
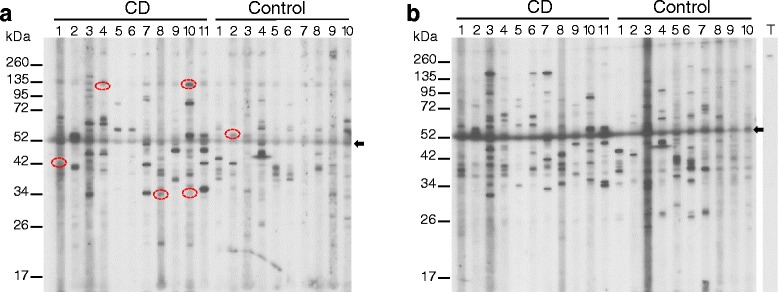
**CD patient serum binds to human cerebellar and cerebral cortex protein extracts in a heterogeneous manner.** Western blots of human brain protein extracts were probed for cross-reacting antibodies with CD and control patient serum. Normal human cerebellar **(a)** and cerebral cortex extracts **(b)** were probed with patient serum for cross-reacting IgG antibodies. CD and control patient serum bound to numerous bands with varying intensity. The circled bands indicate immunoreactive proteins that are unique to the cerebellum immunoreactive to CD but not control serum. Non-identical twins, CD4 and CD5, have immunoreactive patterns different from each other. The arrow heads at ~52 kDa in both panels indicate IgG heavy chain detected by the secondary rabbit anti-human IgG Ab. The rabbit TG6 antibody only detected a >260 kDa band in the cerebral cortex **(b)**. T = anti-TG6 antibody.

Additional Western blotting was performed on total protein extracts of human cerebellum (Figure 3a) and cerebral cortex (Figure 3b) for the presence of autoantibodies in CD patient serum. Similarly to the mouse tissue Westerns, both CD and healthy control samples produced a complex heterogeneous immunoreactive banding profile. Once again, CD4 and 5 – identical twins – produced different patterns of immunoreactivity. Contaminating IgG heavy chain of 55 kDa in both tissue sample extracts was detected by the goat anti-human IgG secondary antibody. Cerebellar-specific immunoreactive proteins were identified in CD patients 1, 4, 8 and 10. Serum from patient CD1 detected a protein of Mr40 kDa, that from CD4 detected faintly a 130 kDa protein, that from CD8 a 34 kDa protein and that from CD10 detected 34 and 135 kDa proteins. The 135 kDa protein detected by CD10 is detected in all other sera but much less intensely (Figure 3a). The bands detected by CD 4 and 8 also appear to be present in Western blots of some healthy control sera. Comparison of CD and control immunoreactive profiles to human cerebellar extracts did not identify proteins that were present in several CD patients but absent in healthy controls, and revealed a different pattern from that seen with mouse cerebellar extracts (Table 1). The TG6 antibody was unable to detect either the 71 or the 79 kDa isoforms in the 500 ug human cerebral cortex total protein extract loaded on the gel (Figure 3b). The TG6 antibody only detected a high MW band of >260 kDa. The TG6 Ab was tested on a separate Western blot of mouse and human cerebellar total protein extracts loaded at the same protein concentration but without preboiling and was found to detect very faint bands of 64, 35 and 32 kDa in mouse and 66 and 32 kDa in human (data not shown).

### CD patient serum does not react to a common cerebellar antigen in mouse or monkey sections

**Figure 4:**
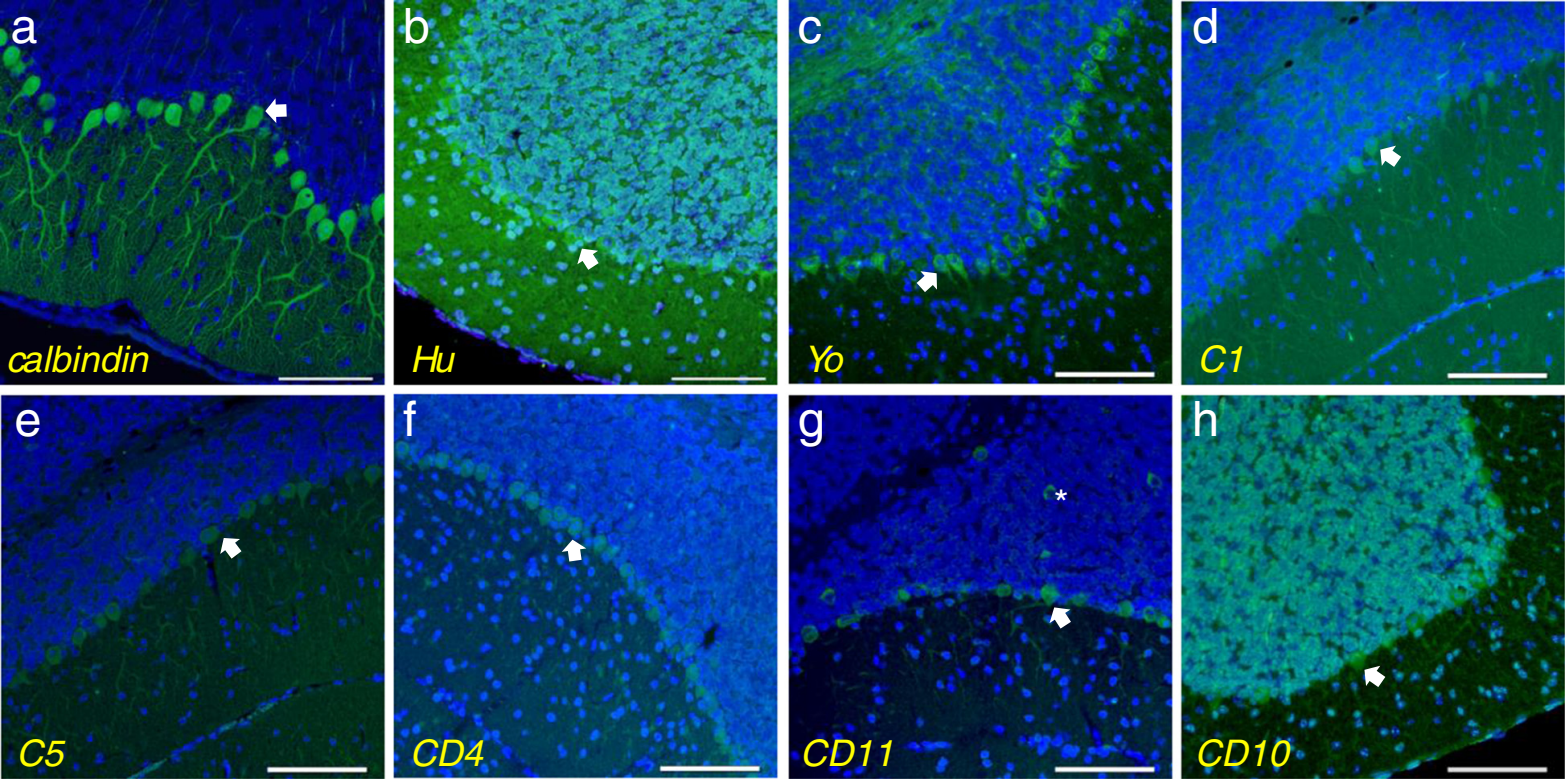
**Mouse cerebellar sections were immunostained with control and CD patient serum for cross-reacting IgG antibodies.** Calbindin staining **(a)** was used as a control to identify PC somata and dendrites. Anti-Hu human auto-immune serum positive control **(b)** demonstrates nuclear immunoreactivity of PCs and granular cells. (Light- blue nuclei indicate co-localisation with DAPI). Anti-Yo auto-immune positive control **(c)** immunostained the cytoplasm of PCs in a granular pattern. Typical control patient immunostaining pattern is demonstrated by serum from C1 **(d)** and C5 **(e)** showing non-specific immunostaining of PCs and non-specific granular layer staining. Representative images of typical CD patient serum immunostaining PCs are shown with CD4 **(f)** and CD11 **(g)**. CD11 **(g)** also demonstrated immunoreactivity to a subset of interneurons. Serum from patient CD10 **(h)** had unique immunoreactivity to all cerebellar nucleoli as well as the cytoplasm of PCs. Arrows indicate PCs. Asterisk indicates interneurons. Scale bar =100 μm.

Mouse cerebellar sections were immunostained with CD patient sera for the presence of cerebellar autoantibodies. Background staining, which consisted of varying levels of diffusely stained PCs, granular cell layer and white matter, was detected in sections probed with secondary Ab only (data not shown). Three different positive controls were also tested: anti-calbindin immunostaining (Figure 4a) showed the normal morphology of the cerebellum – specifically PC somata and dendrites; anti-Hu autoimmune serum bound to the nuclei of PCs and granular cell layer neurons as expected [16] (Figure 4b); anti-Yo autoimmune serum produced coarse granular immunostaining of the PC cytoplasm, as previously reported [17] (Figure 4c). Control human serum from control subjects 1–10 had the same pattern of immunoreactivity as secondary antibody alone, but with a slight increase in PC, molecular layer and granular cell layer immunoreactivity (Figure 4d and 4e). With the exception of patient CD10, the remaining ten CD patients had a similar pattern of immunoreactivity to the healthy controls (Figure 4f), with the PCs showing diffuse immunoreactivity and both molecular and granular cell layers immunostaining to varying degrees. Sera from three CD patients (CD2, 7, 11) and one control (C4) appeared to immunoreact to a subset of interneurons (Figure 4g). A single CD patient, CD10, had a different pattern of immunoreactivity from the other CD patients; the serum immunostained nucleoli of PCs, molecular and granular layer neurons (Figure 4h).

**Figure 5:**
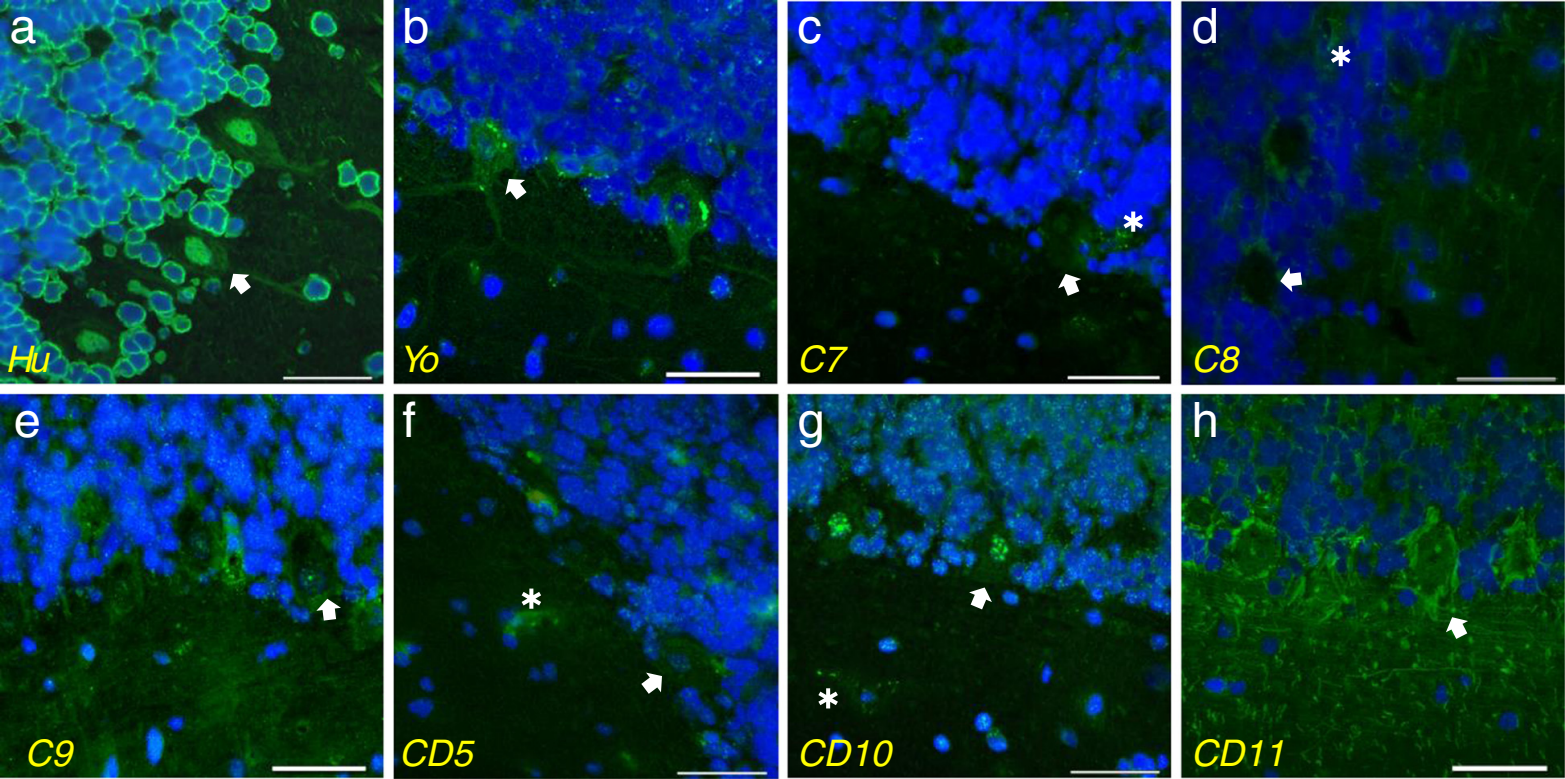
**Cerebellar monkey brain sections were stained with serum from control and CD patients for cross-reacting IgG antibodies.** Anti-Hu human auto-immune serum positive control **(a)** immunostained the nuclei of PCs and granular cells. Anti-Yo **(b)** auto-immune serum showed slight immunoreactivity to PC cytoplasm in a granular pattern. Most non-CD control serum showed immunoreactivity of PCs and endothelial cells as demonstrated by patients C7 **(c)** and C8 **(d)**. Control patient C9 **(e)** differed from the other controls with punctate immunoreactivity to the nucleus of PCs and granule cells. A representative image of CD patient serum with light immunoreactivity of PCs is shown in panel **f** (CD5). Serum from patient CD10 **(g)** stained the nucleoli of all cells of the cerebellum including PCs. CD11 patient serum **(h)** showed basket-like immmunostaining surrounding the PCs. Arrows indicate PCs and asterisk indicates endothelial staining. Scale bar =50 μm.

Monkey cerebellar sections were also used to detect autoantibodies to cerebellar antigens in CD serum. Anti-Hu autoimmune serum, used as a positive control, reacted with the nucleus of PC and granular cell layer neurons, as expected (Figure 5a). The anti-Yo autoimmune serum positive control produced the typical coarse granular immunostaining of the PCs only (Figure 5b). Most healthy controls showed slight immunoreactivity to PCs and presumed endothelial cells (Figure 5c and 5d). Serum from a single healthy control (C9) showed punctate immunoreactivity to the nuclei of PCs, granular cells and molecular layer cells (Figure 5e), which was different from that of anti-Hu (Figure 5a) and patient CD10 (Figure 5g). Figure 5f shows the typical immunoreactivity pattern of nine of the eleven CD patients, with slight immunostaining of PCs and the granular cell layer, as well as presumed endothelial cells. Sera from two patients, CD10 (Figure 5g) and CD11 (Figure 5h), each reacted differently to the cerebellum than those from the other nine CD patients. As in the mouse cerebellum (Figure 4h), CD10 showed punctate immunoreactivity to the nuclei of all cells of the cerebellum (Figure 5g). Serum from this CD patient also reacted to the nuclei of all cells in rat intestine and liver tissue sections (data not shown). Serum from patient CD11 showed a strong basket-like immunoreactivity pattern surrounding the PCs (Figure 5h), in contradistinction to the pattern seen in mice (Figure 4g). As noted, CD11 patient sera had basket-like immunoreactivity surrounding the PCs which resembled that of anti-GAD antibodies [18]. We tested 9/11 CD patients’ sera for anti-GAD, including patient CD 11, and only patient CD9 was positive with a titre of >2,000 U/ml. The remaining eight had a titre of <0.6 U/ml (reference range <5 U/ml).

With respect to those CD and control sera not showing a distinct pattern of immunoreactivity, a blinded observer failed to distinguish between the intensity or pattern of immunoreactivity of sera from CD 1–9 *vs.* controls 1–9.

## Discussion

“Gluten ataxia”, marked by anti-gliadin antibody positivity [2] and possibly relating to anti-tissue transglutaminase-6 antibodies [9], has been advanced as the most common cause of sporadic progressive ataxia [3]. We sought to determine whether there was a common antigen, found in cerebellum but not cerebrum, presumably recognised by anti-gliadin and/or anti-tissue transglutaminase antibodies, in newly diagnosed patients with coeliac disease.

Newly-diagnosed subjects were chosen as their antibody titres were likely to be high, and indeed this was the case. Gliadin is, however, antigenically complex, and the CD subjects demonstrated a broad array of patterns of anti-gliadin reactivity (Figure 1d); many bands are not common across all patients and several are unique. If the relevant cerebellar antigen(s) in gluten ataxia were pathogenic target(s) via cross-reactivity of various anti-gliadin antibodies, it would therefore not be surprising that we found no uniform pattern of cerebellar antigen recognition on Western blotting or immunohistochemistry.

It remains possible that cross-reaction between gliadin and cerebellar antigens involves a gliadin and cerebellar epitope not recognised in our CD subjects, and perhaps unrelated to gut disease [1]. Thus, perhaps only a subset of patients with CD develop antibodies to a unique gliadin epitope that cross-reacts with an accessible (cell surface) cerebellar epitope, and are themselves pathogenic. In indirect support of this, our results illustrate the heterogeneous nature of anti-gliadin antibodies. Tissue transglutaminase-6 has been proposed as the target cerebellar antigen in this regard, being found extracellularly as well as intracellularly in the cerebellum [10]. This is conceivable despite our results – all our CD subjects were selected on the basis of gut rather than of cerebellar disease (although ataxia was not an exclusion criterion), whereas only 6-11% of CD patients have clinically evident ataxia and/or neuropathy [7,19]. Any such antigen would presumably need to be expressed in cerebellum but not cerebrum, as gluten ataxia is typically a pure ataxic syndrome, rather than a heterogeneous one also encompassing limbic encephalitis and/or myelitis as is seen, for example, with anti-Hu disease. It is worth noting, however, that none of the CD sera detected antigens matching the Mr of that identified by the anti-tissue transglutaminase-6 antibody. (More direct testing of CD sera against purified anti-tissue transglutaminase-6 was not performed due to lack of availability of the latter).

Another possibility is that patients susceptible to gluten ataxia may develop pathogenic anticerebellar antibodies in response to a subclinical cerebellar insult with unmasking of cerebellar epitopes cross-reactive with gliadin. Our study would be unable to confirm or refute this possibility, although the lack of a common CD-specific band in Western blots of lysed cerebellar tissue would argue against it. The increased prevalence of anti-gliadin antibodies in those symptomatic with hereditary ataxias [15] is consistent with this otherwise unlikely-seeming hypothesis. Against this is our failure to find a common cerebellar antigen on Western blotting with CD sera, or even a consistent pattern of immunoreactivity on immunohistochemistry, both procedures that should uncover cerebellar epitopes concealed in normal live individuals.

Several other points are worth noting. First, the pattern of immunorecognition of cerebellar-only and of cerebral antigens on Western blotting is appreciably different in mouse compared with that in humans. This suggests that mouse models of passively transferred gluten ataxia [11] may not necessarily be appropriate, and that this difference may have contributed to our previous failure to produce cerebellar damage by active immunisation in HLA-transgenic mice [20]. Second, the anti-neural patterns of immunoreactivity in the identical twin pair on Western blotting were quite different, despite an apparently identical pattern of antigliadin activity, arguing that response to a cerebellar antigen (such as anti-tissue transglutaminase-6) in gluten ataxia is not solely dependent on genetic predisposition. Lastly, sera from one CD subject (11) showed immunoreactivity surrounding PCs in a basket pattern; it seems from this that even antibodies recognising possibly extracellular cerebellar antigens do not necessarily result in ataxia. The basket-like pattern surrounding PCs was not due to anti-GAD antibodies which were only detected in the sera of patient CD 9.

## Conclusion

In summary, the pattern of antigen reactivity in CD is complex and variable for both gliadin and for neural (and specifically for cerebellar) antigens, and is not solely genetically determined. The pattern of immunoreactivity is to some extent species-specific; studies on mice may be misleading. Caution is appropriate in interpreting antibody characterisation studies in gluten ataxia: even apparently positive immunocytochemistry with a cell-surface pattern of reactivity may not be relevant.

Drawbacks of this study include its performance on unselected recently diagnosed CD subjects, none of whom displayed obvious ataxia, and the lack of direct anti-tissue transglutaminase-6 immunoreactivity testing. Advantages include the short duration of treatment prior to testing (ensuring high titres), the careful confirmation of the diagnosis of CD, the use of two different species in each of two different methods of assessing immunoreactivity, and the serendipitous inclusion of an identical twin pair.

## Methods

### Subjects

Eleven subjects with newly-diagnosed coeliac disease were recruited. Their demographic characteristics were as follows: M:F = 3:8; mean age 45 ± 20 years (range 19–77); mean time elapsed between diagnosis and study 5.5 ± 2.9 weeks (range 1.5-11.5). Two subjects (#4 and #5) were identical twins.

Diagnosis was made on small bowel biopsy in all subjects, and in each case confirmatory HLA testing supported the diagnosis, showing all to be *HLA*-DQB_1_*02 positive. In the seven in whom the test results were available, anti-tissue transglutaminase antibodies IgA were also positive.

All underwent ICARS (International Cooperative Ataxia Rating Scale) assessment [21], which has been shown to have high inter-rater reliability [22]. Mean ICARS score was 5.5/100 ± 6 (range 2–15). All were within normal limits of ≤9 [22,23] apart from one subject aged 77 with pre-existing typical essential tremor at 7.5 Hz; subtracting this subject’s tremor-contaminated scores reduced his ICARS score to 5.

For comparison, a convenience sample consisting of 10 healthy, asymptomatic hospital and laboratory workers and subject’s spouses was utilised, with the following characteristics: M:F 5:5; mean age 33 ± 10 years. All were anti-tissue transglutaminase IgA-negative.

Sera was collected from CD and healthy control subjects, and stored at −20/-70°C prior to testing. Control human brain tissue for Western blotting was obtained from the Victorian Brain Bank Network, Melbourne. The study was conducted with the approval of the Alfred Hospital and Monash University Human Research Ethics Committee (Project Numbers: 69/07 and 2007/0498MC).

### ELISA

Serum IgA, IgG- and combined IgA,G,M-class anti-gliadin titres were measured by an in-house-developed indirect enzyme-linked immunosorbent assay (ELISA) as previously described [20]. Briefly, high binding ELISA plates (Greiner) were coated with 500 ng of non-deamidated gliadin (Vital Diagnostics). Patient serum was serially diluted and added to plates in duplicates. Bound antibody was detected with rabbit anti-human IgA, IgG or IgA,G,M horseradish peroxidase (HRP)-conjugated antibodies (DAKO, Australia). Bound antibodies were detected with SigmaFAST OPD substrate (Sigma) and the absorbance was measured using a multi-well plate reader read at 492 nm. Adjusted optical densities (ODs) were calculated by subtracting gliadin-coated wells’ OD values from uncoated wells’ OD values.

An anti-GAD ELISA was performed on 9/11 CD patients by the Royal Melbourne Hospital Pathology service, there being insufficient sera available from identical twins CD4 and CD5 for this assay.

### Western blot

Serum samples were tested for the presence of antibodies to cerebellar and cerebral cortical antigens using a Western blot assay. Cerebellum and cerebral cortex samples from normal humans and C57BL/6 mice were homogenised on ice in lysis buffer (20 mM Tris, 5 mM EDTA, and 1% Nonidet P-40 with a protease inhibitor cocktail (Roche), pH 7.5) followed by sonication for 15 seconds at 5 watts (Qsonica). Tissue homogenates were centrifuged at 20,000 g, 4°C for 10 minutes and the supernatant collected for analysis. Protein concentration was measured with the microBCA assay (Pierce) using bovine serum albumin (BSA) as the standard. 500 ug of total protein (10.9 ug/mm) was separated using a 13% SDS-PAGE separating gel followed by transfer to a 0.22 um pore nitrocellulose membrane (Biorad). The membrane was blocked in 5% skim milk powder in TBS-Tween 20 for 1 hour at room temperature. The membrane was clamped with a Surfblot 30 channel device (Idea Scientific) and patient or control serum (diluted 1:250, 1:500 or 1:1000), rabbit anti-TG6 (1:200; Abcam) and rabbit anti-gliadin (1:1000; Sigma) were applied to separate channels and incubated overnight at 4°C. The membranes were washed in TBS-Tween-20 for 3 h followed by incubation with goat anti-human IgG HRP-conjugated antibody (1:60, 000; Abcam) or swine anti-rabbit-HRP (1:4000; Dako), as appropriate for the respective primary antibodies, for 1 h. Chemiluminescent detection was performed using the Lightning Fast Plus system (Perkin Elmer) according to the manufacturer’s instructions.

### Immunohistochemistry

Mouse cerebellar sections were obtained from HLA-DQ2 transgenic mice [24] as previously described [20]. The 7 μm thick sections were deparaffinised and antigens were un-masked by heating in a microwave with the sections immersed in 10 mM Tris, 1 mM EDTA, 0.05% v/v Tween 20 buffer, pH 9.0. The sections were blocked for 30 min in 0.1% BSA, 2% goat serum in PBS. Patient or control serum (1:500), human anti-Hu (neat; Immco; as a positive control for autoantibodies to Purkinje cells (PCs)), human anti-Yo (neat; Immco; as a positive control for cytoplasmic-specific staining of PCs), and mouse anti-calbindin (1:200; Abcam; as a positive control for Purkinje cell dendrites) were diluted in blocking buffer and incubated overnight at 4 C. The sections were washed in PBS and then fluorescently labelled with secondary antibodies diluted in blocking buffer; goat anti-human AlexaFuor 488 (1:200; Invitrogen), and goat anti-mouse AlexaFluor 488 (1:200; Invitrogen) or goat anti-mouse AlexaFluor 546 (1:200; Invitrogen). The sections were mounted with Vectashield (Vector Labs) containing DAPI as a nuclear counterstain. For primate cerebellar sections (Immco Diagnostics) the same staining protocol was performed, omitting the deparaffinisation and antigen un-masking steps. Each experiment also included negative controls in which primary antibodies were omitted. Immunofluorescent images were taken with an Olympus BX50 microscope. Images of stained sections were assessed for cell types independently by a blinded observer, with respect to positivity above background and pattern of immunoreactivity.
